# Effects of Estrogen and Estrogen Receptors on Transcriptomes of HepG2 Cells: A Preliminary Study Using RNA Sequencing

**DOI:** 10.1155/2018/5789127

**Published:** 2018-10-28

**Authors:** Minqian Shen, Jingyi Cao, Haifei Shi

**Affiliations:** Department of Biology, Miami University, 700 E. High St., Oxford, OH, USA

## Abstract

Men have a much higher incidence of hepatocellular carcinoma (HCC), the predominant form of *liver cancer*, than women, suggesting that estrogens play a protective role in liver cancer development and progression. To begin to understand the potential mechanisms of estrogens' inhibitory effects on HCC development, RNA sequencing was used to generate comprehensive global transcriptome profiles of the human HCC-derived HepG2 cell line following treatment of vehicle (control), estradiol (E2), estrogen receptor alpha- (ER*α*-) specific agonist 1,3,5-tris(4-hydroxyphenyl)-4-propyl-1H-pyrazole (PPT), or ER*β*-specific agonist 2,3-bis(4-hydroxyphenyl)-propionitrile (DPN) using a small set of cells. Gene ontology (GO) analysis identified increased expression of genes involved in the biological process (BP) of response to different stimuli and metabolic processes by E2 and ER agonists, which enhanced molecular function (MF) in various enzyme activities and chemical bindings. Kyoto Encyclopedia of Genes and Genomes (KEGG) functional pathway analysis indicated enhanced pathways associated with carbohydrate metabolism, complement and coagulation cascades, and HIF-1 signaling pathway by E2 and ER agonists. GO analysis also identified decreased expression of genes by E2, PPT, and DPN involved in BP related to the cell cycle and cell division, which reduced MF in activity of multiple enzymes and microtubule activity. KEGG analysis indicated that E2, PPT, and DPN suppressed pathways associated with the cell cycle; E2 and PPT suppressed pathways associated with chemical carcinogenesis and drug metabolism, and DPN suppressed DNA replication, recombination, and repair. Collectively, these differentially expressed genes across HepG2 cell transcriptome involving cellular and metabolic processes by E2 and ER agonists provided mechanistic insight into protective effects of estrogens in HCC development.

## 1. Introduction

Hepatocellular carcinoma (HCC), a common type of primary liver cancer, is one of the most malignant cancers, with increasing incidence and mortality rate worldwide [1–3]. Epidemiologic studies indicate sex dimorphic HCC incidence that is three- to five-fold higher in men compared with women in all groups examined. Additionally, among HCC patients, women have better prognosis with higher survival rates than men [4–9]. Such sex disparity suggests that estrogens may play protective roles in HCC development and progression [4, 7, 10].

Estrogen effects are not limited only to regulate reproductive physiology and function but also to regulate many other important nonreproductive functions, including cell growth, proliferation, apoptosis, immune responses, and metabolism. The liver is one of the estrogen-targeted organs. Estrogens play important roles in regulating cell proliferation and functions in the liver, leading to many sex differences in its gene expression, mitochondrial function, and activity of carbohydrate and lipid metabolic enzymes [10].

Findings from animal research did not clarify the role of estrogens in liver carcinogenesis. Depending on the type of estrogens and animal models, the reported effects are controversial, although the majority of the literature supports estrogens' protective roles in liver cancer. Some studies have reported promotional effects of synthetic estrogens ethinylestradiol in oral contraceptive [11–13] and diethylstilbestrol [14] on liver cancer growth. According to this line of research, it seems that HCC risk would be greater in women because women are exposed to more synthetic estrogens than men for contraception or to prevent complications of pregnancy, which is contradictory to the fact reported by epidemiological studies that HCC incidence is much lower in women than in men. The literature also suggests an association between increased liver cancer and antiestrogen or reduced estrogen levels. Specifically, feeding female rats a diet containing antiestrogen tamoxifen promotes liver tumor growth and induces HCC in these rats [15–17]. Additionally, ovariectomized female mice with reduced endogenous levels of estrogens develop greater numbers of liver tumors compared with intact female mice [18–21]. These studies collectively support the idea that estrogens suppress liver carcinogenesis.

Estrogens act on the nuclear estrogen receptors (ERs) ER*α* and ER*β*, both of which are expressed in diseased liver tissues from HCC specimens [22–26]. ER*α* and ER*β* are ligand-activated transcription factors composed of several domains for hormone binding, DNA binding, and transcriptional activation. Estrogen-ER complex binds to estrogen responsive element of DNA and works as a transcriptional factor that regulates gene expression. The roles of ERs in HCC are complex. Previous studies have reported decreased ER*α* gene expression in human HCC-derived HepG2 cells with hepatitis B virus infection [27, 28] and in liver tumor tissue of HCC patients [29, 30]. Furthermore, Hishida et al. performed a genome-wide analysis in HCC patient samples and identified ER*α* as a candidate tumor suppressor gene [31]. We have reported that estradiol (E2), the predominant and biological active form of estrogens in nonpregnant, premenopausal female subjects, and ER agonists inhibit HepG2 cell proliferation and stimulate apoptosis *in vitro* [32]. Additionally, E2 and ER agonists have been reported to suppress the progression of tumor growth, fibrosis, and HCC carcinogenesis *in vivo* [25, 33, 34]. These studies suggest that the suppression of the ER signaling pathway triggers tumorigenesis leading to HCC, while the activation of ERs reduces HCC. Although this evidence strongly indicates that estrogens and ER signaling have protective effects on HCC pathogenesis, the underlying molecular mechanism largely remains to be elucidated. To understand the potential molecular mechanisms of estrogen and ERs in HCC, RNA sequencing (RNA-Seq) was used to generate comprehensive global transcriptome profiles of HepG2, the most commonly studied human HCC cell line, following treatment of vehicle (control), estradiol (E2), ER*α*-specific agonist PPT, or ER*β*-specific agonist DPN. We hypothesized that E2 and ER agonists would induce an anticancer transcriptomic profile in HepG2 cells.

We searched PubMed using (RNA sequencing OR RNA seq OR sequencing OR microarray) AND (estrogen OR estradiol) AND (HepG2 OR liver cancer OR hepatocellular carcinoma OR liver carcinoma). Majority of these references have studied different types of cancer other than HCC, including breast, ovarian, testicular, uterine endometrial, and bladder cancers, and many of these studies have identified estrogen as a key player. However, both an *in vivo* animal model and *in vitro* cell culture analyses indicate that genetic and genomic regulation by estrogens and ER agonists is highly cell type- and tissue type-specific [35–38]. Thus, transcriptional responses to estrogens and ER agonists in HCC are expected to be quite different from other cancer types. To our knowledge, this is the first study that investigated the effects of E2 and ER agonists in HCC global transcriptome analysis using RNA-Seq. Our findings indicated that HepG2 cells treated with E2, ER*α*-, or ER*β*-specific agonist suppressed cell growth by changing genes related to cell cycles, proliferation, apoptosis, metabolism, and responses to various stimuli. Identifying roles of estrogens and ERs would provide comprehensive understanding of estrogenic mechanisms in HCC development and shed light on potential treatments for liver cancer patients.

## 2. Materials and Methods

### 2.1. Cell Culture

HepG2, a human HCC-derived cell line, was originally obtained from the American Type Culture Collection (ATCC, Manassas, VA, USA) and authenticated using short tandem repeat analysis by ATCC. HepG2 cells were maintained in phenol red-free DMEM supplemented with 10% (*v*/*v*) heat-inactivated and charcoal-stripped FBS, 1% antibiotics of 50 U/ml penicillin, and 50 *μ*g/ml streptomycin (Invitrogen, Grand Island, NY) at 37°C in a humidified atmosphere of 5% CO_2_ and 95% air. The initial cell concentration was 1 × 10^5^/ml. When cells were 70%–80% confluent, culture medium was starved in low serum (0.1% *v*/*v* FBS) for 16 h prior to experiments.

To examine the roles of E2 and specific ERs in growth and transcriptome of HepG2 cells, cells of the control group were treated with 1 *μ*M dimethyl sulphoxide (DMSO; control; *n* = 3) that does not affect gene expression, a serial concentration of water soluble 17*β*-estradiol (E2; 1 *μ*M; Sigma-Aldrich, St. Louis, MO, USA; *n* = 3), ER*α* selective agonist 4,4′,4^″^-(4-propyl-[1H]-pyrazole-1,3,5-triyl)trisphenol (PPT, 1 *μ*M; Fisher, Waltham, MA, USA; *n* = 3), and ER*β* selective agonist 2,3-bis(4-hydroxyphenyl)-propionitrile (DPN; 1 *μ*M; Fisher, Waltham, MA, USA; *n* = 3). The doses of these chemicals are based on our preliminary dose curve analysis and are commonly used in liver cancer cell culture studies [32, 39]. All chemicals were first dissolved in DMSO and then diluted to final concentration using cell culture medium. Cells were harvested 48 hours after treatment, a time period with growth differences among treatment in HepG2 cells and optimal for determining gene expression. Notably, this study should be considered as a preliminary study due to the relatively small sample size.

### 2.2. Cell Counting, Proliferation, and Apoptosis

The numbers of cells with diameters within a 6–50 *μ*m range were measured using a TC10TM automated cell counter (Bio-Rad, Hercules, CA, USA). Cell proliferation was indicated by bromodeoxyuridine (BrdU) incorporation analysis using an enzyme-linked immunosorbent assay (ELISA) kit (Millipore Corporation, Billerica, MA, USA). Cell apoptosis was indicated by an apoptosis marker cleaved cysteine-aspartic acid protease (caspase) 3, a critical executioner in apoptotic cells responsible for proteolytic cleavage of many key proteins [40], using a Western blotting detection kit (Cell Signaling, Danvers, MA, USA).

### 2.3. RNA Isolation and Quality Control

Total RNA was isolated from ~10^6^ HepG2 cultured cells using an RNeasy Mini Kit (Qiagen, Foster City, CA, USA) following the manufacturer's instructions. The RNA samples were monitored by gel electrophoresis, and their concentrations were quantified using a NanoDrop 2000 Spectrophotometer (Thermo Scientific, Wilmington, DE, USA). Concentrated RNA samples were submitted to the Genomics, Epigenomics and Sequencing Core at the University of Cincinnati (http://med.uc.edu/eh/cores/genomics). High quality of RNA samples with an RNA integrity number (RIN) above 9 was used for library preparation.

### 2.4. RNA-Seq

The RNA-Seq was performed according to the manufacturer's protocols as well as standardized protocols developed by the Genomics, Epigenomics and Sequencing Core facility at the University of Cincinnati. An automated, strand-specific library preparation method was employed using PrepX RNA-Seq samples and a library preparation kit (WaferGen Biosystems, Fremont, CA, USA) with Apollo 324 NGS automated library prep system (WaferGen Biosystems). Briefly, the isolated polyA-RNA from 1 *μ*g total RNA input was fragmented to 200 nucleotides with RNase III, adaptor-ligated to single-stranded RNA, and converted into complementary DNA (cDNA) using superscript III reverse transcriptase (LifeTech, Grand Island, NY, USA), followed by automated purification using Agencourt AMPure XP beads (Beckman Coulter, Indianapolis, IN). The cDNA libraries were amplified with 12 PCR cycles using the universal and index-specific primer, purified by AMPure XP beads in Apollo 324 system, and the quality and yield of purified library were checked by Bioanalyzer (Agilent, Santa Clara, CA, USA) using a DNA high sensitivity chip. The library concentration for clustering was measured by Kapa Library Quantification kit (Kapa Biosystems, Woburn, MA, USA) using ABI's 9700HT real-time PCR system (Thermo Fisher). Individually indexed and compatible libraries were proportionally pooled for clustering in the cBot system (Illumina, San Diego, CA, USA). The libraries were then sequenced as single-end 50 bp on the Illumina HiSeq 2000 system.

### 2.5. Transcriptome and Functional Analysis

Approximately 25 million sequence reads were retrieved from each sample and were aligned to the human genome (ENSEMBL GRCh38.p10) using the standard Illumina sequence analysis pipeline. Differential gene expression was analyzed using R package DESeq2 by the Laboratory for Statistical Genomics and Systems Biology at the University of Cincinnati. All the genes produced by the DESeq2 differential analysis were ranked by the log2 fold change values (Tables S1–S3). The expression levels of genes were analyzed to identify differentially expressed genes (|log2 fold change| > 1.5; *P*_adj_ < 0.01), because some genes with small fold changes could be statistically significant but may not be promising candidates for further investigation. The sequence data were deposited at Gene Expression Omnibus (GEO) https://www.ncbi.nlm.nih.gov/geo/info/seq.html, a publicly available database. The accession number is GSE112983.

Gene ontology (GO) enrichment analysis was performed using ClueGO (http://apps.cytoscape.org/apps/cluego) [41]. GO annotates significantly expressed genes to a biological process (BP), molecular function (MF), and Kyoto Encyclopedia of Genes and Genomes (KEGG) terms, and assigns genes to functional pathways [42]. Terms with a *P* value < 0.05 were considered significant.

### 2.6. Quantitative Real-Time PCR

Total RNA (1 *μ*g) isolated from HepG2 cells treated with DMSO (control), E2, PPT, or DPN (*n* = 3 for each treatment group) was reverse transcribed into cDNA using a cDNA synthesis kit (Bio-Rad, Hercules, CA). The primers were synthesized by Integrated DNA Technologies (San Jose, CA). Relative expression of three differentially expressed genes indicated by RNA-Seq and known to be regulated by estrogens, *PPARG*, *SOCS3*, and *IL6R*, was measured in this preliminary study. Other identified differentially expressed genes will be validated in the future. *ACTB* was used as a reference gene, since *ACTB* mRNA level did not vary among groups with different treatments according to RNA-Seq analysis. *ACTB* forward primer was GTG GGG CGC CCC AGG CAC CA, and reverse primer was GTC CTT AAT GTC ACG CAC GAT TTC. *PPARG* forward primer was TCT GGC CCA ACT TTG GG, and reverse primer was CTT CAC AAG CAT GAA CTC CA. *SOCS3* forward primer was GGA GTT CCT GGA CCA GTA CG, and reverse primer was TTC TTG TGC TTG TGC CAT GT. *IL6R* forward primer was CAG CTG AGA ACG AGG TGT CC, and reverse primer was GCA GCT TCC ACG TCT TGA. Quantitative real-time PCR was carried out using SYBR green master mixes and an iCycler (Bio-Rad, Hercules, CA). Amplified products were confirmed via gel electrophoresis and melt curve analysis. Results were generated from triplicate experiments. Relative quantification of gene expression was normalized using the housekeeping gene *ACTB*, calculated by the 2^−ΔΔCt^ method [43], and presented using the control group as 100%.

### 2.7. Statistical Analyses

Cell number, proliferation, apoptosis, and gene expression from quantitative PCR data were presented as mean ± SEM and were compared using one-way analysis of variance (ANOVA) followed by Tukey's multiple comparison post hoc test (GraphPad Prism 7; La Jolla, CA, USA) to compare different treatment effects. *P* values < 0.05 was considered statistically significant. The differentially expressed genes among treatment groups were analyzed using R package DESeq2 by the Laboratory for Statistical Genomics and Systems Biology at the University of Cincinnati with a |log2 fold change| > 1.5 and *P*_adj_ < 0.01 considered to be statistically significant.

## 3. Results

### 3.1. Effects of E2 and ER Agonists on HepG2 Cell Number, Proliferation, and Apoptosis

**Figure 1:**
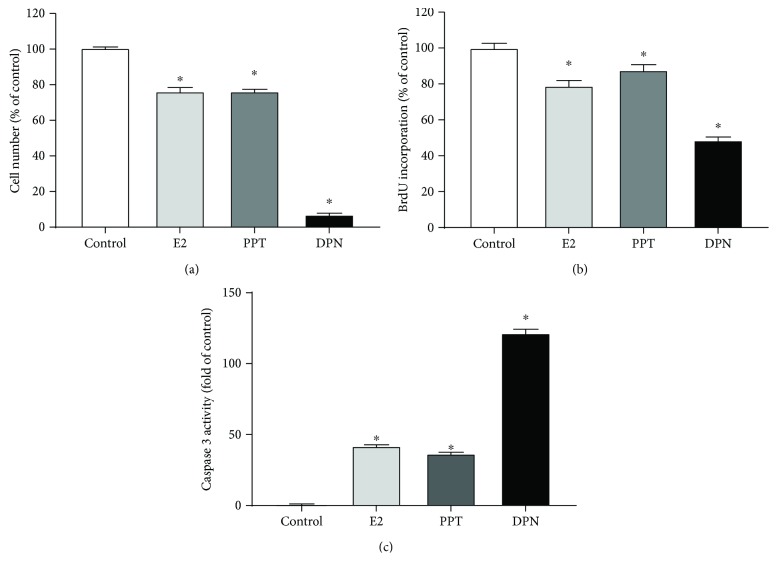
Effects of 17*β*-estradiol (E2), estrogen receptor (ER) *α* agonist PPT, and ER*β* agonist DPN on cell number (a), proliferation indicated by BrdU incorporation (b), and apoptosis indicated by caspase 3 activity (c) of HepG2 cells. ^∗^Significantly different comparing to vehicle-treated (control) HepG2 cells (*P* < 0.05).

E2 and ER agonists significantly reduced the HepG2 cell number comparing with the control group (Figure 1(a)), consistent with our previous finding [32]. Specifically, E2 and ER*α* agonist PPT similarly reduced cell number by 75.90 ± 2.09% and 76.36 ± 1.05%, respectively; while ER*β* agonist DPN further reduced cell number to 7.03 ± 0.82% compared to the control group. Cell numbers were determined by cell proliferation and apoptosis, both of which were assessed. Cell proliferation mirrored the results of cell numbers among treatment groups. E2 and ER agonists significantly suppressed cell proliferation, indicated by lowered BrdU incorporation, compared with the vehicle-treated cells (Figure 1(b)). Specifically, E2 and ER*α* agonist PPT significantly reduced BrdU incorporation to 79.02 ± 2.95% and 87.75 ± 2.95%, respectively, and ER*β* agonist DPN further decreased BrdU incorporation to 48.70 ± 1.77% of control cells with vehicle treatment. E2 and ER agonists also significantly induced cell apoptosis, indicated by increased activity of caspase 3, compared with the control group (Figure 1(c)). Similar to the effects of E2 and ER agonists on cell proliferation, ER*β* agonist DPN had the greatest effect on the induction of apoptosis. Specifically, E2 and ER*α* agonist PPT significantly promoted apoptosis by 41.96 ± 0.84 and 36.62 ± 0.94 folds, respectively, and ER*β* agonist DPN further promoted apoptosis by 121.52 ± 2.82 folds compared with the cells of the control group. These results indicated that E2 and ER-specific agonists suppressed HepG2 cell growth via suppressing proliferation and promoting apoptosis, with ER*β*-specific agonist having greater effects than E2 and ER*α*-specific agonist.

### 3.2. Effects of E2 and ER Agonists on HepG2 Cell Global Transcriptome

Regulation of the target genes in response to E2, PPT, or DPN treatment was analyzed using RNA-Seq as an approach for transcriptome-wide gene expression profiling. The transcriptome analysis revealed 956 upregulated genes and 380 downregulated genes by E2 treatment (Table S1), 242 upregulated genes and 397 downregulated genes by ER*α*-specific agonist PPT treatment (Table S2), and 254 upregulated genes and 271 downregulated genes by ER*β*-specific agonist DPN treatment (Table S3), compared to the control treatment.

**Figure 2:**
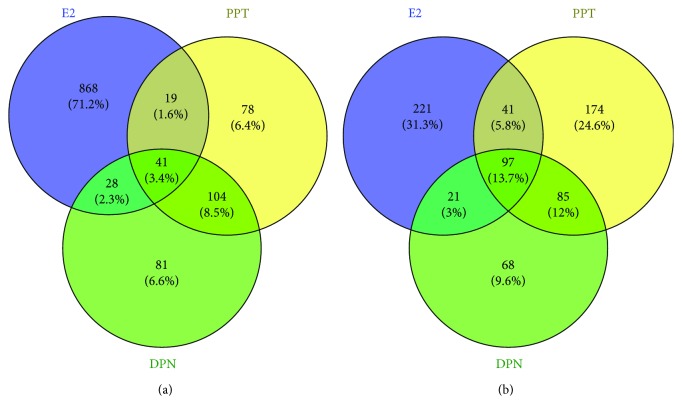
Effects of 17*β*-estradiol (E2), estrogen receptor (ER) *α* agonist PPT, and ER*β* agonist DPN on differential gene expression of HepG2 cells detected using RNA sequencing. Venn diagrams indicate the unique and shared (a) upregulated and (b) downregulated genes of different treatments.

Among the upregulated genes, 868 unique genes were upregulated by E2 treatment, 78 unique genes were upregulated by PPT treatment, and 81 unique genes were upregulated by DPN treatment. 60 common genes were upregulated by E2 and PPT, 69 common genes were upregulated by E2 and DPN, and 145 common genes were upregulated by PPT and DPN. Additionally, the Venn diagram analysis identified 41 upregulated genes being commonly regulated across all three treatments (Figure 2(a); Table S4).

Among the downregulated genes, 221 unique genes were downregulated by E2 treatment, 174 unique genes were downregulated by PPT treatment, and 68 unique genes were downregulated by DPN treatment. 138 common genes were downregulated by E2 and PPT, 118 common genes were downregulated by E2 and DPN, and 182 common genes were downregulated by PPT and DPN. Additionally, there were 97 common genes downregulated by all three treatments (Figure 2(b); Table S4).

### 3.3. Effects of E2 and ER Agonists on Upregulated Pathways in HepG2 Cell

We then investigated the biological roles of the differentially expressed genes of HepG2 cells treated with E2 or ER agonists using GO analysis and grouped into BP, MF, and KEGG functional pathway enrichment analysis.

**Figure 3:**
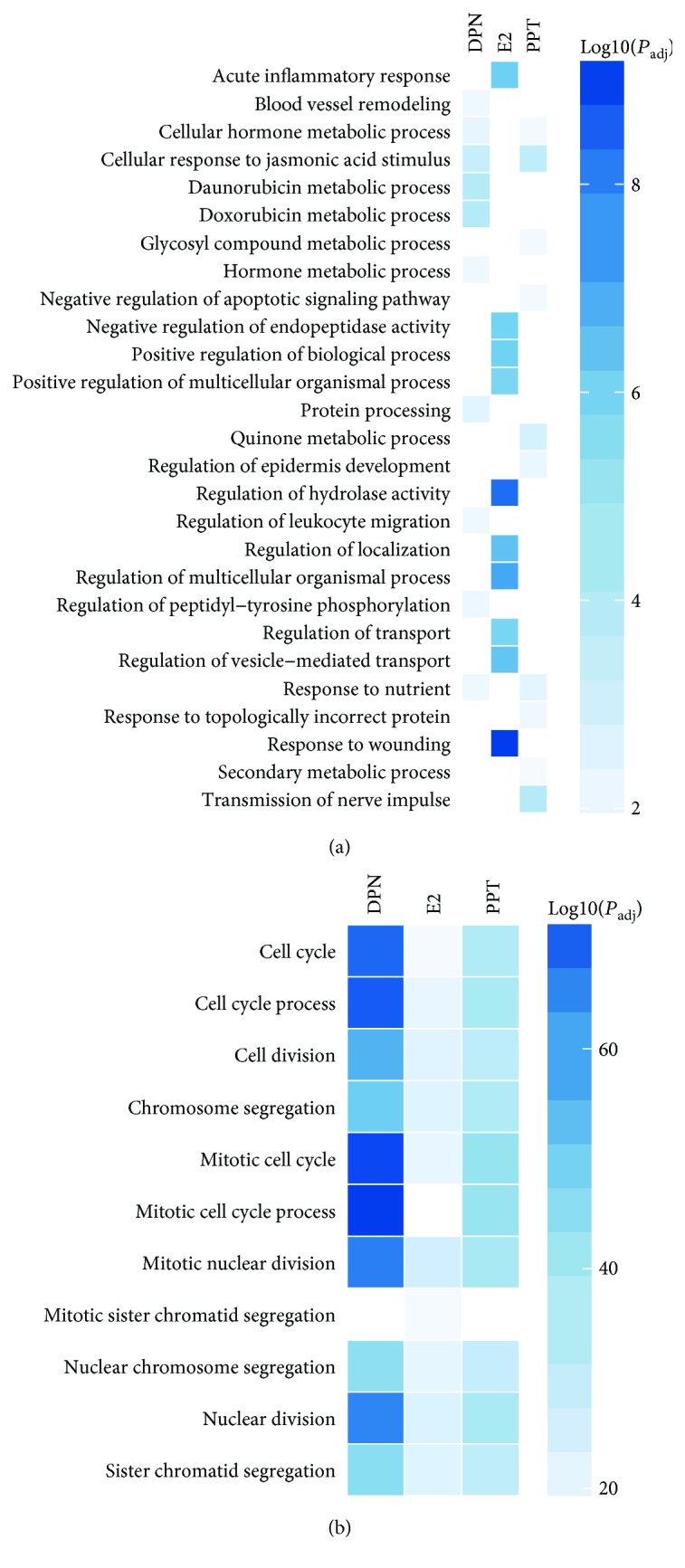
Heat map showing and comparing the top gene ontology (GO) biological process (BP) annotation terms among 17*β*-estradiol (E2), estrogen receptor (ER) *α* agonist PPT, and ER*β* agonist DPN treatments of HepG2 cells. Enrichment analysis of differentially expressed genes was performed on the list of (a) upregulated and (b) downregulated genes compared with vehicle-treated HepG2 cells.

**Figure 4:**
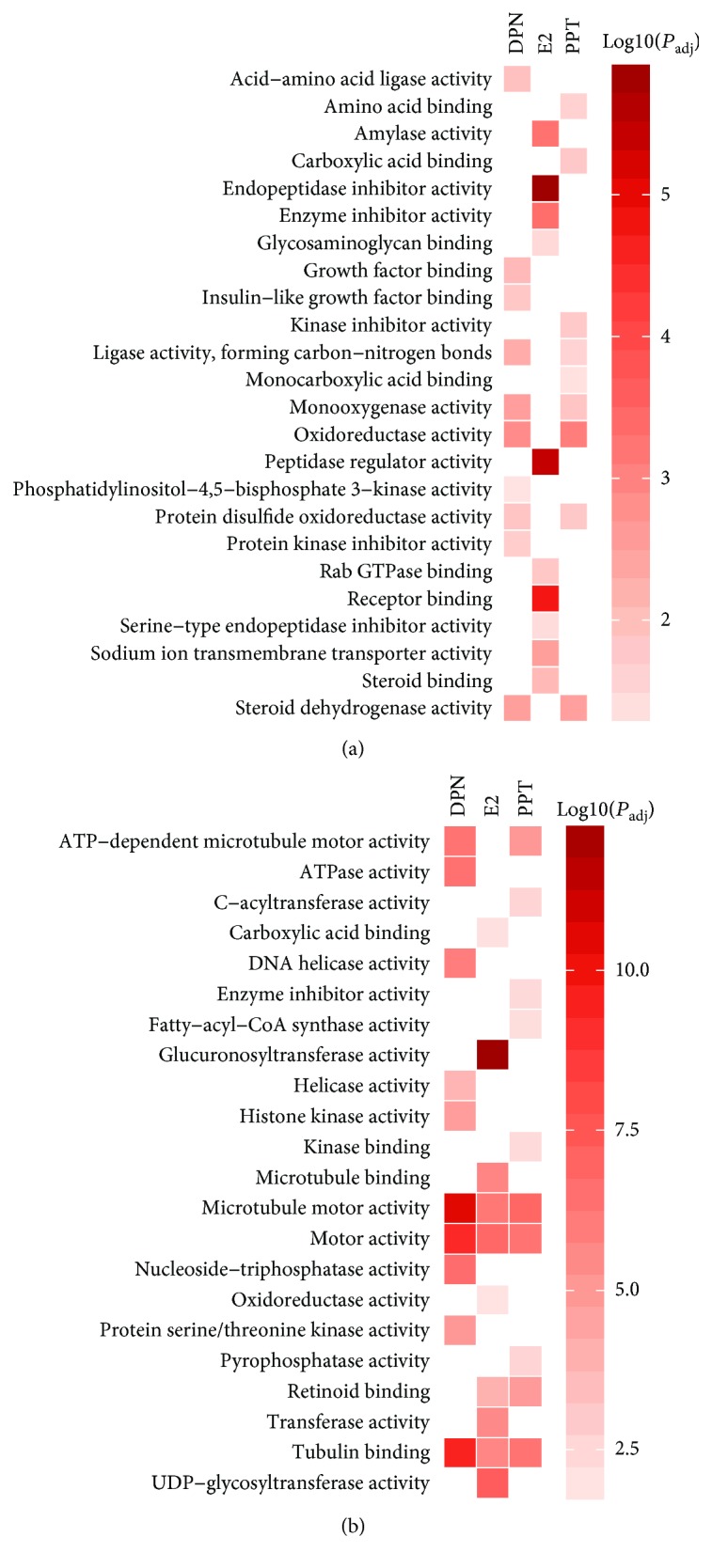
Heat map showing and comparing the top gene ontology (GO) molecular function (MF) annotation terms among 17*β*-estradiol (E2), estrogen receptor (ER) *α* agonist PPT, and ER*β* agonist DPN treatments of HepG2 cells. Enrichment analysis of differentially expressed genes was performed on the list of (a) upregulated and (b) downregulated genes compared with vehicle-treated HepG2 cells.

**Figure 5:**
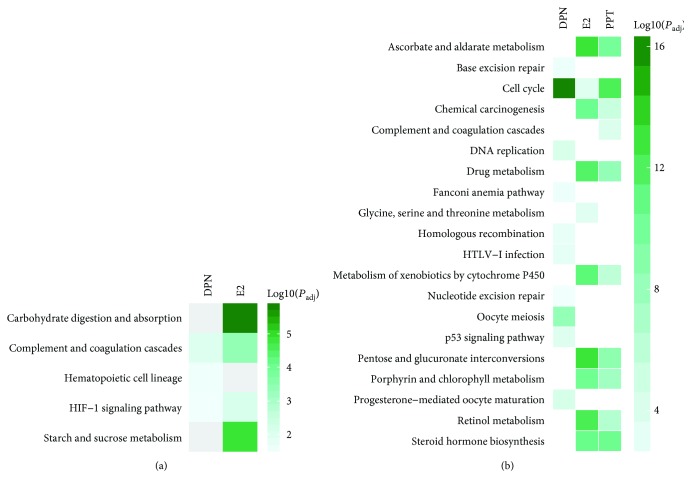
Heat map showing and comparing the top Kyoto Encyclopedia of Genes and Genomes (KEGG) annotation terms among 17*β*-estradiol (E2), estrogen receptor (ER) *α* agonist PPT, and ER*β* agonist DPN treatments of HepG2 cells. Enrichment analysis of differentially expressed genes was performed on the list of (a) upregulated and (b) downregulated genes compared with vehicle-treated HepG2 cells.

GO analysis identified increased expression of genes involved in BP related to responses to wounding and acute inflammation, regulation of biological and multicellular organismal processes, localization, transport, and activities of hydrolase and endopeptidase by E2 (Figure 3(a); Table S1). The upregulated genes were enriched in MF annotations linked to activities of enzymes, their regulators, and Na^+^ transporter, as well as bindings of steroid, receptor, GAPase, and glycosaminoglycan by E2 (Figure 4(a); Table S1). These upregulated genes associated with KEGG pathways linked to carbohydrate metabolism, complement and coagulation cascades, and hypoxia-inducible factor-1 (HIF-1) signaling pathway were enhanced by E2 treatment (Figure 5(a); Table S1).

GO analysis identified upregulated genes involved in BP related to nerve impulse transmission, cellular response to jasmonic acid stimulus, negative regulation of apoptotic signaling pathway, regulation of epidermis development, and metabolic processes of hormone and various nutrients by ER*α* agonist PPT (Figure 3(a); Table S2), which enhanced MF in activities of oxidoreductase, steroid dehydrogenase, monooxygenase, carbon-nitrogen bond ligase, and kinase inhibitor, and bindings of carboxylic acid, monocarboxylic acid, and amino acid (Figure 4(a); Table S2).

The upregulated genes by ER*β* agonist DPN were involved in BP related to the regulation of the metabolic process, response to jasmonic acid stimulus and nutrient, peptidyl-tyrosine phosphorylation, leukocyte migration, blood vessel remodeling, and protein processing (Figure 3(a); Table S3), which enhanced MF in activities of oxidoreductase, monooxygenase, protein kinase regulator, steroid dehydrogenase, and ligase, as well as growth factor binding (Figure 4(a); Table S3). The top enhanced pathways by DPN treatment were complement and coagulation cascades, hematopoietic cell lineage, and HIF-1 signaling (Figure 5(a); Table S3).

Notably, E2 treatment had most evident impact among three treatment groups on upregulated genes that are involved in cell metabolism and function (Figures 3(a), 4(a), and 5(a)).

### 3.4. Effects of E2 and ER Agonists on HepG2 Cell Downregulated Pathways

GO analysis identified commonly downregulated genes among E2-, PPT-, and DPN-treated HepG2 cells involved in BP related to the cell cycle, cell and nuclear division, and chromosome and chromatid segregation (Figure 3(b)), which mostly reduced MF in microtubule activity and tubulin binding (Figure 4(b)). KEGG functional pathway analysis indicated that pathways associated with the cell cycle were suppressed by all E2, PPT, and DPN treatments. Additionally, E2 and PPT suppressed pathways associated with chemical carcinogenesis, steroid hormone biosynthesis, and drug metabolism, whereas DPN suppressed pathways associated with DNA replication, homologous recombination, nucleotide and base excision repair, and p53 signaling pathway, as well as oocyte meiosis and maturation (Figure 5(b)). Furthermore, the 97 commonly downregulated genes by all three treatments were associated with MF in motor activity, tubulin binding, and cyclin-dependent protein kinase activity. Cell cycle, p53 signaling pathway, and oocyte meiosis and maturation were commonly represented KEGG pathways suppressed by all three treatments (Table S4). Among these three treatments, DPN most robustly downregulated genes regulating the cell cycle and division (Figures 3(b), 4(b), and 5(b)), consistent with the lowest cell numbers and proliferation observed in the cells treated with DPN (Figure 1).

### 3.5. Validation of RNA-Seq Results Using Quantitative PCR

Three differentially expressed genes from E2- or ER agonist-treated cells identified by RNA-Seq, peroxisome proliferator-activated receptor gamma (*PPARG*, GeneID 5468), suppressor of cytokine signaling 3 (*SOCS3*, GeneID 9021), and interleukin 6 receptor (*IL6R*, GeneID 3570) were validated using quantitative PCR. The mRNA levels of housekeeping gene *β*-actin (*ACTB*, GeneID 60) were similar among all groups, and thus, *ACTB* was used as a housekeeping gene.

**Figure 6:**
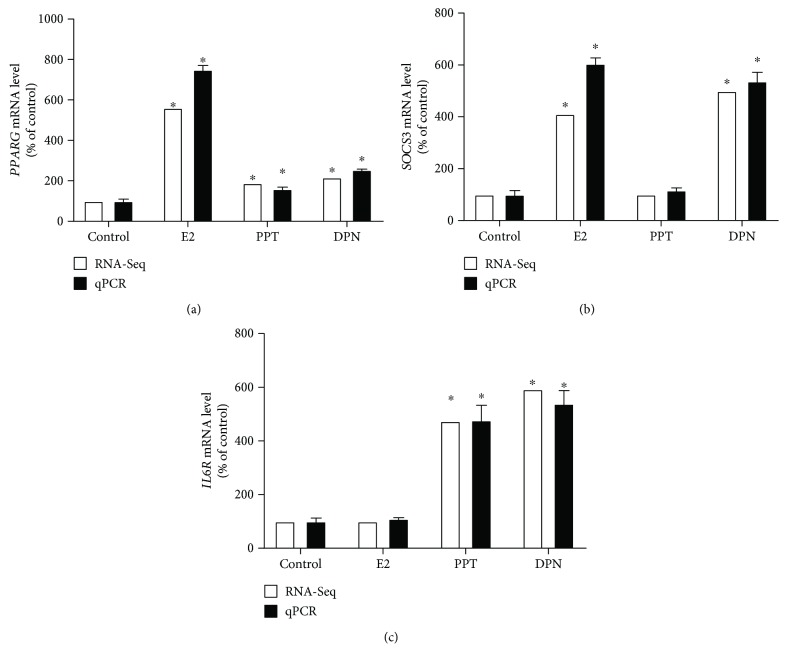
Validation of expression of peroxisome proliferator-activated receptor gamma (*PPARG*; (a)), suppressor of cytokine signaling 3 (*SOCS3*; (b)), and interleukin 6 receptor (*IL6R*; (c)) using quantitative RT-PCR. *β*-Actin (*ACTB*) was used as a reference gene. ^∗^Significantly different comparing to vehicle-treated (control) HepG2 cells (*P* < 0.05).

Based on the RNA-Seq results, the increases in the expression of *PPARG* were 5.60-fold by E2 (log2 fold change = 2.49; *P*_adj_ = 2.3*E* − 52), 1.88-fold by PPT (log2 fold change = 0.91; *P*_adj_ = 1.15*E* − 06), and 2.17-fold by DPN (log2 fold change = 1.12; *P*_adj_ = 2.46*E* − 11) compared with the control group. Quantitative PCR indicated a similar trend, and increased expressions of *PPARG* were 7.50 ± 0.20-fold by E2 (*P* < 0.05), 1.60 ± 0.09-fold by PPT (*P* < 0.05), and 2.54 ± 0.04-fold by DPN (*P* < 0.05; Figure 6(a)). RNA-Seq data also revealed that the expression of *SOCS3* was significantly elevated to 4.11-fold by E2 (log2 fold change = 2.04; *P*_adj_ = 1.76*E* − 08) and to 5.00-fold by DPN (log2 fold change = 2.32; *P*_adj_ = 4.84*E* − 13), but was not changed by PPT, compared with the control group. Quantitative PCR indicated significant increase in the expression of *SOCS3* to 6.05 ± 0.22 (*P* < 0.05) and 5.37 ± 0.35 (*P* < 0.05) folds by E2 and DPN, respectively, and nonsignificant change by PPT treatment (1.16 ± 0.10, *P* > 0.05), compared with the control group (Figure 6(b)). RNA-Seq data also indicated significant increases in the expression of *IL6R* to 4.74-fold by PPT (log2 fold change = 2.24; *P*_adj_ = 5.26*E* − 97) and to 5.93-fold by DPN (log2 fold change = 2.57; *P*_adj_ = 5.71*E* − 194), but no change by E2, compared with the control group. Quantitative PCR indicated similar trends that changed the expression of *IL6R* to 1.10 ± 0.04 (*P* > 0.05), 4.77 ± 0.56 (*P* < 0.05), and 5.39 ± 0.49 (*P* < 0.05) folds by E2, PPT, and DPN, respectively (Figure 6(c)). Therefore, gene expression levels obtained using RNA-Seq and quantitative PCR methods were in good agreement with small variation in the magnitude of expression.

## 4. Discussion

This preliminary study demonstrated that E2 and ER agonists reduced HCC HepG2 cell growth via suppressing proliferation and promoting apoptosis and identified hundreds of differentially expressed genes in a small set of HepG2 cells treated with E2 or a specific ER agonist using RNA-Seq. While RNA-Seq is a powerful tool for exploring differentially expressed genes, the expression of identified genes and the production of corresponding proteins need to be validated and cellular functions suggested by pathway analyses need to be confirmed.

The incidence of HCC has increased worldwide. In the United States, HCC has become the fastest rising cause of cancer-related deaths. Epidemiology studies from different regions of the world show that men have a much higher incidence of HCC and greater mortality than women [8], suggesting that estrogens may have protective roles in HCC. Although many *in vivo* and *in vitro* HCC models have been studied, whether estrogens play a protective or destructive role in HCC is still under debate. It is widely accepted that estrogens exert carcinogenic effects via activating their receptors [44, 45]. Indeed, estrogens and ERs have been implicated to promote carcinogenic effects via activating proliferation and mitotic division in reproductive cancers, especially breast, ovarian, and uterine endometrial cancers in females [46, 47], and in these cancer cell lines [48]. It is noteworthy that the carcinogenic effect by estrogens is tissue type-specific. For example, one study has reported that estrogen replacement therapy in menopausal women increases the risks of gallbladder, breast, endometrial, and urinary bladder cancers, while lowers the risks of other types of cancers, including liver, colon, and rectum cancers [49]. The present study confirmed that E2 and ER agonists reduced HCC HepG2 cell growth by suppressing proliferation and promoting apoptosis (Figure 1). Therefore, estrogens may work through distinct mechanisms in different types of tissues and cells to regulate divergent sets of genes. It is possible to develop drugs that confer estrogenic effects in liver tissue without incurring harmful effects in other tissues such as breast, uterine, and ovarian tissues.

Some specific genes affected by estrogens in HCC models have been evaluated in multiple basic and clinical research studies and have been identified as molecular markers targeting specific cell signaling pathways altered by estrogens. The approaches applied in these studies however are only able to test a limited number of genes, presenting incomplete snapshots of entire mechanistic pathways affected by E2 and ER agonists in liver cancer development. The current study implemented a global, broad-content transcriptomic RNA-Seq analysis that provided a big picture of unbiased characterization of complex transcriptional responses of HCC HepG2 cells treated with E2 and ER agonists. Common gene expression signatures across all treatment groups, as well as genes uniquely responsive to E2 and ER-specific agonists, were analyzed by GO terms in BP, MF, and KEGG pathways. Across all treatments, upregulated pathways were predominantly associated with metabolic processes linked to lipid and carbohydrate metabolism that were most evidently affected by E2 treatment, and downregulated genes were mainly involved in the cell cycle, proliferation, and apoptosis that were most robustly affected by DPN treatment.

Several pathways linked to cell cycle, proliferation, growth, and apoptosis were identified in the present study. One of the most prominent changes was that all treatments of E2 and ER agonists downregulated genes responsible for cell cycle progression from G_1_ to S phase and from G2 to M phase (Tables S1–S3). These genes involved in the G_1_/S and G2/M transition are critical for cell cycle progression [50]; consequently, pathways related to the cell cycle and division, meiotic cell cycles, chromosome and chromatic organization were downregulated (Figure 5(b)). Besides suppressing cell cycle progression, E2 and ER agonists also affected the expression of growth-related genes to inhibit cancer cell proliferation. A few negative growth-regulatory genes were identified differentially expressed by RNA-Seq. For example, E2 upregulated growth arrest and DNA damage-inducible beta (*GADD45B*, GeneID 4616), whose activation stimulates apoptosis and inhibits proliferation in HCC [51], and both E2 and DPN downregulated teratocarcinoma-derived growth factor 1 (*TDGF1*, GeneID 6997) that promotes liver cancer development [52].

Growth factors, along with their receptors and binding proteins, play diverse and complex roles. The intricate cross talk among various growth factor signaling pathways in HCC growth involves insulin-like growth factors (IGF), platelet-derived growth factors (PDGF), transforming growth factors (TGF), fibroblast growth factors (FGF), and others [53]. IGF system includes IGF1 and IGF2, IGF receptors, and IGF-binding proteins IGFBP1–7. In circulation, IGFBPs bind to IGFs with high affinity along with the acid-labile subunit (ALS) to form the IGF-IGFBP-ALS complex, which is unable to cross endothelia of vessels and is confined to the circulation. This leads to reduced availability of IGFs to their receptors, suppressed IGF signaling, and thus inhibited mitotic, proliferative, and invasive effects in HCC development [54, 55]. The present RNA-Seq data revealed enhanced expression of several players in the IGF signaling pathway, including *IGFBP1* (GeneID 3484) across all treatments, *IGFBP2* (GeneID 3485) by E2, and *IGFBP3* (GeneID 3486) by E2 and DPN. Intriguingly, our data suggested that E2 downregulated *IGFBP7* (GeneID 3490) but upregulated *IGF2* (GeneID 3481), which contradicted its suppressive effects in HCC.

IGFBPs have been implicated in suppressing the transcription of tumorigenesis-promoting genes [56, 57], such as early growth response proteins (EGRs) [58] and EGR target genes PDGF and FGF [55]. In support of this view, the present RNA-Seq data indicated that *EGR1* (GeneID 1958), *EGR2* (GeneID 1959), *PDGFC* (GeneID 56034), and *PDGFD* (GeneID 80310) were all downregulated by E2. The literature has demonstrated cooperative interaction between PDGFs and TGFs. Specifically, PDGFC activates TGF *β* signaling [59], and TGF *β* activates PDGF signaling [60]. Current RNA-Seq data were consistent with the literature showing that *TGFB3* (GeneID 7043) and TGF*β* receptor 3 (*TGFBR3*, GeneID 3490) were downregulated by ER*α* agonist PPT. Additionally, FGF receptor 2 (*FGFR2*, GeneID 2263) was downregulated by PPT and DPN. However, several FGFs (*FGF11*, GeneID 2256 and *FGF17*, GeneID 8822) and TGFs (*TGFA*, GeneID 7039 and *TGFB1*, GeneID 7040) were upregulated by E2 treatment. Such data did not support the antiproliferative and suppressive role of E2 in cell division indicated by reduced BrdU incorporation (Figure 1(b)) or KEGG pathway analysis (Table S1). This seemingly contradictory finding may reflect the complexity and genetic redundancy of the interplay between estrogens and growth factor signaling pathways in HCC development.

E2 and DPN upregulated the hypoxia-inducible factor 1 (HIF-1) signaling pathway. Due to rapid proliferation of tumor cells and inadequate oxygen supply, hypoxia is typically presented in the microenvironment of tumor cells [61]. Hypoxic cells adaptively activate HIF-1 signaling to induce angiogenic factors and increase perfusion [62]. Although the current RNA-Seq did not find differences in the expression of HIF1 or HIF2, it revealed that a few angiogenic factors were upregulated. For example, E2 upregulated vascular endothelial growth factors (*VEGFA*, GeneID 7422 and *VEGFB*, GeneID 7423); E2 and DPN upregulated epidermal growth factor receptor (*EGFR*, GeneID 1956), and DPN upregulated heparin-binding epidermal growth factor-like growth factor (*HBEGF*, GeneID 1839). Both tumor-promoting and tumor-suppressing roles of the HIF-1 signaling pathway have been documented. For example, elevated levels of HIF-1 are presented in tumor tissues and the suppression of HIF-1 impairs cancer growth, indicating oncogenic effects of HIF-1 signaling [63, 64]. In contrast, activation or constant expression of HIF-1 reduces tumor growth [65, 66], HIF-1 deficiency promotes tumor survival [67, 68], and overexpression of HIF-1 suppresses tumor growth while the inhibition of HIF-1 enhances tumor growth [62, 69]. The dual effects of HIF-1 signaling would allow optimal response to hypoxia by different types of cells. Koshiji and Huang have proposed “stop-and-go” strategy [70]. Briefly, for some types of tumors, HIF signaling increases angiogenesis and oxygen supply for glycolysis that provides energy for tumor cells to survive; while for other types of tumors, the induction of cell cycle arrest and apoptosis is the best way to survive, especially when oxygen supply is limited.

E2 and ER agonists also upregulated genes that promote tumor cell death. This is consistent with increased activity of caspase 3, whose sequential activation plays a central role in the execution phase of cell apoptosis of HepG2 cells treated with E2, PPT, and DPN (Figure 1(c)). Tumor necrosis factor (TNF) superfamily member (*TNFSF14*, GeneID 8740) and TNF receptor superfamily members (*TNFRSF11A*, GeneID 8792 and *TNFRSF25*, GeneID 8718) were upregulated by E2. Expression of *TNFSF14*, however, was downregulated by ER*α* agonist PPT. Additionally, TNF superfamily members (*TNFSF10*, GeneID 8743 and *TNFSF9*, GeneID 8744) as well as tumor suppressor (*TSG1*, GeneID 643432) were upregulated by ER*β* agonist DPN. Both ER*α* and ER*β* agonists also upregulated tumor suppressor candidate 3 (*TUSC3*, GeneID 7991) and downregulated tumor protein p73 (*TP73*, GeneID 7161) (Tables S1–S3). Two homologous proteins p53 and p73 have functional similarity and structural resemblance. Both p53 and p73 induce cell cycle arrest, DNA damage, and apoptosis [71]. Expression of *TP73* in human cancers is complex. Different from the well-studied tumor suppressor gene *TP53*, *TP73* is rarely mutated in cancers. Analyses of breast tumors from patients and cell lines derived from breast tumors show a higher expression of *TP73* compared with normal tissues and cells [72, 73]. Additionally, mouse models with *TP53* deficiency [74] but not with *TP73* deficiency [75] show spontaneous tumorigenic and carcinogenic phenotypes. These findings imply that, different from the tumor suppressor p53, tumor protein p73 could function as a complex oncoprotein [71].

Interestingly, RNA-Seq data identified differentially expressed genes linked to other types of cancers than HCC. For example, pituitary tumor-transforming 1 interacting protein (*PTTG1IP*, GeneID 754) was upregulated by E2 treatment, but pituitary tumor-transforming 1 (*PTTG1*; GeneID 9232) was downregulated by E2, PPT, and DPN. Suppression of tumorigenicity 14 colon carcinoma (*ST14*, GeneID 6768) was downregulated by PPT.

When estrogens or ER agonists bind to ERs, the complex translocates into the nucleus and binds to estrogen responsive elements and other transcription factors to regulate target gene transcription. The present RNA-Seq data suggested cross talk between ER and other nuclear receptors. Specifically, E2 treatment upregulated interleukin 3-regulated nuclear factor (*NFIL3*, GeneID 4783), nuclear receptor subfamily 1 group D member 1 (*NR1D1*, GeneID 9572), nuclear receptor subfamily 4 group A member 1 (*NR4A1*, GeneID 3164), while downregulated nuclear receptor subfamily 1 group I member 2 (*NR1I2*, GeneID 8856). PPT downregulated erythroid 2 nuclear factor (*NFE2*, GeneID 4778).

It is noteworthy that PPARs are transcription factors of nuclear receptors. PPARG, a subtype of PPAR superfamily, predominantly regulates metabolic changes associated with lipid and carbohydrate homeostasis that affect cancer cell growth. Specifically, PPARG is responsible for peroxisomal *β*-oxidation of fatty acids in the liver. Upregulation of PPARG in all treatment groups (Figure 6(a)) suggested interference with key regulators of lipid metabolism in HepG2 cells by E2 and ER agonists. PPARG has been reported to play a protective role in some types of cancer but play an oncogenic role in other types of cancer. Specifically, several studies have reported PPARG in HCC prevention and treatment. PPARG ligands induce apoptosis and cell cycle arrest and inhibit proliferation and cell growth in HCC cells [76–79]. Consequently, the activation of PPARG leads to the induction of apoptosis and the inhibition of cell proliferation in human HCC cell lines [76–79]. PPARG expression was enhanced by E2 and to a much lesser degree by ER agonists (Figure 6(a)), indicating that PPARG is highly susceptible to E2 comparing with specific ER agonists. Although E2 is not a PPAR ligand, it indirectly activates PPARs [80]. Existing literature indicates that the cross talk between E2 and PPAR signaling is a cell type-specific event. For example, E2 upregulates PPARG expression in adipocytes [81], but E2 suppresses PPARG transcription via binding competitively to shared transcriptional factors in human breast cancer cells [82]. Consistent with previous findings, we found that PPARG activation in liver cancer cells by E2 and ER agonists inhibits proliferation, induces cell cycle arrest (Figures 1(a) and 3(b)) [78, 83–85], and induces apoptosis through caspase 3 activation (Figure 1(c)) [78, 86].

The present RNA-Seq study also detected altered expressions of functional genes involved in lipid biosynthesis, transportation, and metabolism. Apolipoprotein (APO) gene cluster (APOA1/C3/A4/A5) on human chromosome 11q23 regulates circulating lipid level via influencing incorporation and transporting lipids in the blood [87]. Expressions of *APOC3* (GeneID 345) and *APOA5* (GeneID) were upregulated by E2. The main function of APOLs is to initiate apoptosis and regulate cell death [88]. E2 upregulated *APOL1* (GeneID 8542), *APOL2* (GeneID 23780), and *APOL4* (GeneID 80832), consistent with its apoptotic effects. Low-density lipoprotein (LDL) receptors are present on the cell membrane of liver cells, which enable cholesterol to enter cells to reduce circulating LDL levels. LDL receptor-related proteins (LRPs) expressed on liver cell membrane are members of the LDL receptor family and uptake circulating chylomicron remnants [89]. E2 upregulated LDL receptor class A domain containing 1 (*LDLRAD1*, GeneID 388633), and PPT upregulated *LRP8* (GeneID 7804). DPN upregulated *LRP10* (GeneID 26020) but downregulated *LRP2* (GeneID 4036). The present RNA-Seq data suggested overall enhanced lipid uptake by E2 and ER agonists.

Besides those genes functional in lipid metabolism, some genes associated with glucose metabolism were also changed by E2 and ER agonists. Under hypoxic conditions, cells shift glucose metabolism from more efficient oxidative phosphorylation to less efficient glycolysis, an effect known as the Warburg effect. The present RNA-Seq data demonstrated that E2 upregulated the glycolysis enzymes glucose-6-phosphatase catalytic subunit (*G6PC*, GeneID 2538) and hexokinase 1 (*HK1*, GeneID 3098), as well as a facilitated glucose transporter solute carrier family 2 member 3 (*SLC2A3*, GeneID 6515), indicating increased glucose utilization and transport into cells to maintain energy production.

Altered cell growth, apoptosis, and cell cycle in tumor development correlates with nutrient-deprived microenvironment and induced immune responses. Consistent with our previous finding [32], the expression of *SOCS3* was upregulated by E2 and ER*β* agonist DPN but not by ER*α* agonist PPT (Figure 6(b)), suggesting that estrogenic SOCS-mediated effect against HCC growth is primarily via ER*β*. Altering the hepatic expression of *SOCS3 in vivo* has been reported to regulate signaling of other members of the cytokine superfamily, such as IL6 [90]. Additionally, estrogen has been demonstrated as a key negative regulator of IL6 production in cervical cancer HeLa cells [91]. In the present study, although none of the treatments changed the expression of IL6, the expression of *IL6R* (Gene ID 3570) was upregulated by PPT and DPN (Figure 6(c)). This finding suggested that SOCS3 stimulation by estrogen could regulate IL6 signaling via IL6R. Furthermore, our data indicated that E2 or ER agonist regulated other chemokines. For example, E2 downregulated C-C motif chemokine ligand (CCL) 16 (*CCL16*, GeneID 6360) and PPT downregulated *CCL20* (GeneID 6364), whereas E2 upregulated *CCL26* (GeneID 10344), C-C motif chemokine receptor 7 (*CCR7*, GeneID 1236), and C-X-C motif chemokine ligand 16 (*CXCL16*, GeneID 58191).

To summarize, E2 and ER agonists enhanced the expression of apoptosis-promoting genes coupled with downregulation in proliferative genes, altered lipid and glucose metabolisms, thereby changed the balance between cell death and cell growth in HepG2 cells. Additionally, E2 and ER agonists regulated many HCC-secreting growth factors, angiogenic factors, and inflammatory cytokines, whose signaling pathways cooperatively regulate liver cancer cell growth, proliferation, and progression. HepG2 cell line is the most commonly studied liver cancer cell line. Nevertheless, other HCC cell lines will be studied in future to validate if estrogen effects seen in this study can be generalized to HCC or are specific of HepG2 cell line.

## 5. Conclusions

HCC is one of the most common and deadly cancers worldwide with women having lower incidence, better prognosis, and higher survival rate than men [2, 3]. We previously reported that E2 suppressed HepG2 cell proliferation mainly through ER*β* action [32]. This study explored potential underlying mechanisms integrating metabolic, cellular, and molecular pathways by E2 and its receptor agonists using RNA-Seq transcriptome analysis, a powerful tool that not only helps to understand genetic responses of carcinogenesis but also identifies new biomarkers with diagnostic and predictive values. In conclusion, our findings suggest that estrogens inhibit HepG2 cell growth by suppressing cell proliferation and inducing cell apoptosis via estrogen receptor signaling pathways. Specific genes identified in this study, especially those involved in cell cycle regulation, hold a great therapeutic potential for HCC treatment. Furthermore, the transcriptional landscape defined by RNA-Seq provides a global view of altered gene expression signatures associated with lipid and glucose metabolisms, which could be potential targets for novel liver cancer therapies.

## Data Availability

The sequence data are deposited at a publicly available database Gene Expression Omnibus (GEO) https://www.ncbi.nlm.nih.gov/geo/info/seq.html. The accession number is GSE112983. The full lists of differentially expressed genes and gene ontology terms used to support the findings of this study are included within the supplementary information files Tables S1–S4. All the cell physiology, quantitative PCR, and gene ontology heat map data used to support the findings of this study are included within the article.

## Abbreviations

ACTB: *β*-Actin
ALS: Acid-labile subunit
APO: Apolipoprotein
BP: Biological processes
CCL: C-C motif chemokine ligand
DPN: 2,3-Bis(4-hydroxyphenyl)-propionitrile
E2: Estradiol
EGR: Early growth response protein
ER: Estrogen receptor
FGF: Fibroblast growth factor
G6PC: Glucose-6-phosphatase catalytic subunit
GO: Gene ontology
HBEGF: Heparin-binding epidermal growth factor-like growth factor
HCC: Hepatocellular carcinoma
HIF-1: Hypoxia-inducible factor-1
HK1: Hexokinase 1
IGF: Insulin-like growth factor
IGFBP: Insulin-like growth factor-binding protein
IL6R: Interleukin 6 receptor
KEGG: Kyoto Encyclopedia of Genes and Genomes
LDL: Low-density lipoprotein
MF: Molecular function
PDGF: Platelet-derived growth factor
PPARG: Peroxisome proliferator-activated receptor gamma
PPT: 1,3,5-Tris(4-hydroxyphenyl)-4-propyl-1H-pyrazole
RNA-Seq: RNA sequencing
SOCS3: Suppressor of cytokine signaling 3
TGF: Transforming growth factor
TNF: Tumor necrosis factor
VEGF: Vascular endothelial growth factor.

## Supplementary Materials

Supplementary Materials Table S1: differentially expressed genes between 17*β*-estradiol (E2) and vehicle-treated (control) HepG2 cells, gene ontology (GO) biological process (BP) terms, molecular function (MF) terms, and Kyoto Encyclopedia of Genes and Genomes (KEGG) terms associated with upregulated and downregulated genes. Table S2: differentially expressed genes between estrogen receptor (ER) *α* agonist PPT and vehicle-treated (control) HepG2 cells, gene ontology (GO) biological process (BP) terms, molecular function (MF) terms, and Kyoto Encyclopedia of Genes and Genomes (KEGG) terms associated with upregulated and downregulated genes. Table S3: differentially expressed genes between estrogen receptor (ER) *β* agonist DPN and vehicle-treated (control) HepG2 cells, gene ontology (GO) biological process (BP) terms, molecular function (MF) terms, and Kyoto Encyclopedia of Genes and Genomes (KEGG) terms associated with upregulated and downregulated genes. Table S4: common differentially expressed genes of 17*β*-estradiol (E2), estrogen receptor (ER) *α* agonist PPT, and ER*β* agonist DPN, comparing with vehicle-treated (control) HepG2 cells, gene ontology (GO) biological process (BP) terms, molecular function (MF) terms, and Kyoto Encyclopedia of Genes and Genomes (KEGG) terms associated with common upregulated and downregulated genes.
